# Dietary supplement use in ambulatory cancer patients: a survey on prevalence, motivation and attitudes

**DOI:** 10.1007/s00432-021-03594-7

**Published:** 2021-04-06

**Authors:** Maja Tank, Kristina Franz, Emanuele Cereda, Kristina Norman

**Affiliations:** 1 Medizinisches Versorgungszentrum Tempelhof Oncology, Berlin, Germany; 2 Department of Geriatrics, Charité—Universitätsmedizin Berlin, Corporate Member of Freie Universität Berlin and Humboldt—Universität zu Berlin, Reinickendorfer Str. 61, 13347 Berlin, Germany; 3 Institute of Nutritional Science, University of Potsdam, Potsdam, Germany; 4 Clinical Nutrition and Dietetics Unit, Fondazione IRCCS Policlinico San Matteo, Pavia, Italy; 5 Department of Nutrition and Gerontology, German Institute of Human Nutrition Potsdam—Rehbrücke, Nuthetal, Germany

**Keywords:** Dietary supplements, Dietary habits, Patients with cancer, Nutritional counselling

## Abstract

**Purpose:**

Patients with cancer often believe dietary supplements (DS) such as micronutrients and botanical products to be health supporting and non-toxic despite growing concerns regarding potential pharmacological interactions. Studies on the prevalence of DS use among patients with cancer are heterogeneous and mainly conducted at university-based cancer centers. This survey focused on a particular cancer patient group treated in an ambulatory setting without regular access to professional nutritional counselling.

**Methods:**

Patients with a history of cancer or hematological malignancy were included in this survey. A self-reported questionnaire was used to evaluate the different aspects of DS use, changes in dietary habits and patients’ demographic characteristics.

**Results:**

Almost every second patient reported using DS (47.2%). Women (56.3%), patients with an academic degree (56.0%) and non-smokers (84.8%) were more inclined to use DS. Along with magnesium (16.6%), calcium (14.3%), multivitamins (12.0%) and vitamin C (9.4%), use of herbal supplements (12.6%) was common. Women (84.8% vs. 74.9% of men, *p* =  < 0.001) and patients younger than 65 years (84.4% vs. 77.2% of patients > 65 y, *p* =  0.002) sought dietary advice more often. Support of the immune system was the main reason for DS use (26.4%) and a relevant number of patients (49.6%) reported to have changed their dietary habits following cancer diagnosis.

**Conclusion:**

DS use is common among patients with cancer treated in an ambulatory setting. This finding should encourage oncologists to implement detailed questioning about DS use and dietary habits to prevent potential interactions and offer substantial advice.

## Introduction

Use of dietary supplements (DS) such as vitamins, trace elements, minerals and botanical products has become increasingly popular among patients with cancer, believing DS to be non-toxic and health-supporting and therefore to be used as self-medication (Bailey et al. 2011; Li et al. 2010). Studies estimate the prevalence of DS use ranging from 18 to 95% depending on the patient population, tumor stage and cultural background (Du et al. 2020; Luo and Asher 2018; Molassiotis et al. 2005; Wilkinson and Stevens 2014).

Despite the popularity of DS, the use in disease is controversially discussed among health professionals and scientists due to increasing evidence regarding potential interactions with conventional therapies, leading to either an increase in toxicity or loss of effectiveness. Potential harm is also derived by the fact that patients often do not disclose the use of DS to their doctors (Davis et al. 2012; Levy et al. 2018). Nevertheless, studies show a highly unmet need for a consultation about complementary alternative medicine, and DS in particular (Horneber et al. 2018).

A special feature of the German health system is the decentralized patient care system situated at specialized doctor’s offices. In fact, a significant proportion of patients with cancer in Germany are treated in such ambulatory settings. In contrast to many hospitals and comprehensive cancer centers, they do not offer a routine dietary counselling.

Therefore, in this study we investigated the prevalence of DS use including herbal and botanical supplements in patients with cancer treated in an ambulatory setting with respect to demographic characteristics as well as clinical features such as type of disease, disease duration and type of therapy approach. Moreover, the study aims to assess the attitude of patients towards DS and their motivation for DS use as well to gather information regarding the sources of dietary information in ambulatory patients with cancer.

## Methods

### Study population

A cross-sectional survey was conducted in patients with cancer, seeking care at three ambulatory cancer care centers, between September 2011 and October 2012 and September 2017 and December 2019. Inclusion criteria were a history of solid tumor disease, malignant hematological disease or chronic hematological non-malignant condition and age ≥ 18 years. Patients were classified according to the administered therapy: any kind of oncological medication, best supportive care, surgical intervention only, radiotherapy only and no intervention at all (watch and see strategy). All participants gave written informed consent. The study was reviewed and approved by the Ethics Committee of the Charité University Hospital, Berlin.

### Data collection

To assess different aspects of DS use, a questionnaire was developed containing open-end and closed questions as well as multiple-choice questions, allowing multiple answers. The main part of the questionnaire comprises questions regarding the use of DS, frequency and duration of DS use, type of DS and dosage. Different kinds of vitamins, minerals and trace elements are listed as well as botanical or herbal supplements, special teas and immune-stimulating supplements. The second part focuses on sources of information regarding DS use and patients’ motivation for using DS. Moreover, we asked for changes in dietary habits including preferences or avoidance of certain foods since cancer diagnosis. The last part of the questionnaire asks for demographic and clinical data, such as diagnosis, time since diagnosis, tumor stage, treatment history and comorbidities. The questionnaire was tested in a pre-test involving 20 patients with cancer to prove practicability and comprehensibility—in particular, the understanding of specific terms. The test patients were also interviewed about their DS use (intake, frequency and duration) and asked to bring their products to compare results from the questionnaire and the interview.

### Statistical analysis

All statistical analyses were performed using IBM SPSS Version 23 and 25. For nominal variables, results were described by frequencies (*n*) and percentage (%). *χ*^2^ test and Fisher’s exact test were used to compare categorical parameters. Continuous variables were reported as mean and standard deviation (SD) or median and interquartile range (IQR). Two-group and multiple-group comparisons were performed using the Student’s *t* test and ANOVA or the non-parametric tests Mann–Whitney *U* and Kruskal–Wallis. *p *values below 0.05 were a priori considered statistically significant.

## Results

A total of 1217 (51.3% female) out of 1452 patients (Table 1) completed the questionnaire which reflects an 83.8% response rate. 54.5% of patients suffered from solid cancer with a median duration of disease of 18 months (5–44 months). Colorectal cancer was the most common disease followed by breast cancer and other cancer of the genital organs. 40.1% of patients had advanced or metastatic disease. 39.2% of patients had hematological neoplasms with a median duration of disease of 29 months (8–70 months). Within this group, Non-Hodgkin lymphoma, multiple myeloma and myeloproliferative neoplasm were the most frequently diagnosed. Furthermore, 6.3% reported other chronic hematological non-malignant conditions such as chronic autoimmune thrombocytopenia (2.6%) and monoclonal gammopathy of unknown origin (3.3%) with a median duration of disease of 38 months (16–70 months). Regarding the different therapy strategies, the following distribution pattern was found: 71.9% of patients received some kind of oncological medication, 6.6% reported to be on best supportive care, 4.6% underwent surgical intervention only, 2.4% had radiotherapy only and 12.2% did not receive any kind of intervention at all (watch and see strategy).

**Table 1:** Sociodemographic characteristics and lifestyle factors of the study population

Characteristics	All *n* = 1217	DS users *n* = 574	Discontinued DS use *n* = 55	Non DS users *n* = 585	*p* value
Age (years)	67.6 ± 12.9	68.0 ± 12.5	65.8 ± 13.3	67.4 ± 13.2	0.425
Sex, *n* (%)					
Female	624 (51.3)	324 (56.3)	32 (58.2)	267 (45.6)	0.001
Male	593 (48.7)	251 (43.7)	23 (41.8)	318 (54.4)	
Current living situation, *n* (%)					
Living alone	373 (30.9)	174 (30.6)	16 (29.6)	182 (31.2)	0.958
Living with partner	835 (69.1)	394 (69.4)	38 (70.4)	401 (68.8)	
Education level*, *n* (%)					
Non-academic	993 (82.6)	456 (79.6)	45 (83.3)	492 (85.6)	0.028
Academic	209 (17.4)	117 (20.4)	9 (16.7)	83 (14.4)	
Smoking status**, *n* (%)					
Non smoker	974 (81.2)	480 (84.8)	39 (72.2)	455 (78.6)	0.023
Previous smoker	18 (1.5)	9 (1.6)	1 (1.9)	8 (1.4)	
Current smoker	207 (17.3)	77 (13.6)	14 (25.9)	116 (20.0)	
BMI (kg/m^2^)	25.7 ± 4.8	25.4 ± 4.9	25.4 ± 4.9	25.9 ± 4.6	0.231

DS users were defined as patients who used ≥ 1 DS, regularly. Patients who stopped their DS intake at study entrance were classified in the interrupted DS user group. Data are given as mean ± SD or as absolute values (and %) and were performed using the ANOVA and the χ^2^ test

*DS* dietary supplements, *BMI* body mass index

*Missing values: 15 (1.2%), **missing values: 18 (1.5%)

### Frequency of DS use

Of 1217 patients, 47.2% reported using DS at study entrance, 4.5% of patients had taken DS until recently and 48.1% denied use of DS.

Overall, women and patients with an academic background as well as non-smokers reported using DS more frequently (see Table 1). Participants with chronic hematological non-malignant condition used DS more often than patients with solid tumors or hematological neoplasms as follows: 61.8% vs. 47.3% vs. 45.0%, *p* = 0.035).

Within the group of DS users, 41.5% started DS use after cancer diagnosis whereas 37.1% had used DS regularly and 19.2% occasionally before cancer diagnosis. The majority of patients reported DS use daily (64.8%) and for longer than 12 months (64.5%).

We found sex-specific differences only within the group of patients with solid tumor disease, which was related to the cohort with cancer of the genital tract, including breast cancer (Table 2). Duration of disease differed between DS users and non-users (24.0 months [IQR 7–62] vs. 21.0 months [IQR 6–50], *p* = 0.045), whereas tumor stage (*p* = 0.170) or patients’ reported therapeutic approach did not (*p* = 0.980).

**Table 2:** Type of solid tumor disease and hematological neoplasms according to sex and use of DS (*n* = 1217)

Type of disease	Women			Men			*p* value*
	DS users *n* = 323	Discontinued DS use *n* = 32	Non DS users *n* = 268	DS users *n* = 251	Discontinued DS use *n* = 23	Non DS users *n* = 317	
Solid tumor location, *n* (%)							
Genital tract, breast or prostate	86 (58.5)	4 (2.7)	57 (38.8)	20 (37.7)	3 (5.7)	30 (56.6)	0.022
Lung or respiratory tract	20 (57.1)	3 (8.6)	12 (34.3)	22 (48.9)	0	23 (51.1)	0.060
Gastrointestinal tract	38 (48.7)	7 (9.0)	33 (42.3)	63 (41.7)	7 (4.6)	81 (53.6)	0.164
Hepatobiliary or pancreatic tract	11 (42.3)	4 (15.4)	11 (42.3)	16 (37.2)	1 (2.3)	26 (60.5)	0.086
Kidney or bladder	3 (30.0)	1 (10.0)	6 (60.0)	7 (53.8)	1 (7.7)	5 (38.5)	0.583
Head and neck	2 (66.7)	0	1 (33.3)	8 (50.0)	1 (6.3)	7 (43.8)	1.000
Other	13 (46.4)	1 (3.6)	14 (50.0)	4 (28.6)	1 (7.1)	9 (64.3)	0.395
Hematological neoplasms, *n* (%)							
Non-Hodgkin lymphoma	40 (56.3)	4 (5.6)	27 (38.0)	26 (44.8)	2 (3.4)	30 (51.7)	0.277
Multiple myeloma	21 (45.7)	2 (4.3)	23 (50.0)	26 (55.3)	1 (2.1)	20 (42.6)	0.592
Myeloproliferative neoplasm	20 (38.5)	1 (1.9)	31 (59.6)	13 (32.5)	0	27 (67.5)	0.808
Chronic lymphocytic leukemia	17 (63.0)	0	10 (37.0)	17 (44.7)	2 (5.3)	19 (50.0)	0.304
Myelodysplastic syndrome	10 (37.0)	2 (7.4)	15 (55.6)	9 (34.6)	1 (3.8)	16 (61.5)	1.000
Acute leukemia	6 (60.0)	0	4 (40.0)	5 (29.4)	1 (5.9)	11 (64.7)	0.299
Hodgkin lymphoma	2 (18.2)	1 (9.1)	8 (72.7)	2 (40.0)	0	3 (60.0)	0.698
Chronic hematologic disease	34 (66.7)	2 (3.9)	15 (29.4)	13 (50.0)	2 (7.7)	11 (42.3)	0.362

Data are presented as frequencies (%). Percentages refer to type of disease

*DS* dietary supplements

*p values were calculated between sex with Fisher’s exact test

### Type of DS use

Magnesium and calcium supplementation was most frequently reported followed by herbal and botanical supplements, multivitamins, vitamin D and the vitamin B group, which is shown in summary as well as according to sex and age in Table 3. Only a minority of participants (< 5%) gave detailed dosage information of DS products. Detailed analysis of the reported types of herbal and botanical supplements revealed more than 50 different kinds of products, e.g. curcumin preparations, milk thistle, black cumin oil, St John’s wort, mistletoe, seaweed extract (data not shown).

**Table 3:** Type and frequency of DS use according to sex and age categories in all patients

Types of DS	All *n* = 1217	Sex		*p* value	Age		*p* value
		Women *n* = 624	Men *n* = 593		≤ 65 y *n* = 456	> 65 y *n* = 760	
Vitamins, *n* (%)							
Vitamin D	133 (10.9)	78 (12.5)	55 (9.3)	0.071	45 (9.9)	87 (11.4)	0.392
Vitamin B group	132 (10.8)	76 (12.2)	56 (9.4)	0.125	42 (9.2)	90 (11.8)	0.153
Vitamin C	115 (9.4)	62 (9.9)	53 (8.9)	0.552	43 (9.4)	72 (9.5)	0.980
Vitamin E	41 (3.4)	21 (3.4)	20 (3.4)	0.994	14 (3.1)	27 (3.6)	0.652
Multivitamins, *n* (%)	146 (12.0)	72 (11.5)	74 (12.5)	0.614	57 (12.5)	89 (11.7)	0.682
Micronutrients and trace elements, *n* (%)							
Magnesium	202 (16.6)	105 (16.8)	97 (16.4)	0.826	63 (13.8)	139 (18.3)	0.042
Calcium	174 (14.3)	94 (15.1)	80 (13.5)	0.433	57 (12.5)	117 (15.4)	0.163
Zinc	69 (5.7)	41 (6.6)	28 (4.7)	0.163	24 (5.3)	45 (5.9)	0.631
Iron	62 (5.1)	36 (5.8)	26 (4.4)	0.272	23 (5.0)	39 (5.1)	0.946
Selenium	50 (4.1)	29 (4.6)	21 (3.5)	0.331	27 (5.9)	23 (3.0)	0.014
Immune stimulating supplements, *n* (%)	75 (6.2)	47 (7.5)	28 (4.7)	0.042	34 (7.5)	41 (5.4)	0.148
Omega-3 fatty acids, *n* (%)	69 (5.7)	33 (5.3)	36 (6.1)	0.555	26 (5.7)	43 (5.7)	0.974
Herbal and botanical supplements, *n* (%)	153 (12.6)	102 (16.3)	51 (8.6)	0.001	61 (13.3)	92 (12.2)	0.603
Tea, *n* (%)	200 (16.4)	124 (19.9)	76 (12.8)	0.001	78 (17.1)	122 (16.1)	0.632

Data presented as frequencies (%). *p* values are calculated with *χ*^2^ test. Multiple answers allowed

*DS* dietary supplements

Women reported the use of herbal and botanical supplements, tea and immune-stimulating supplements more often than men. We also found a difference between age groups, as younger patients used selenium more often, and older patients used magnesium more frequently (see Table 3).

### Patients’ attitude and motivation for DS use

**Fig. 1:**
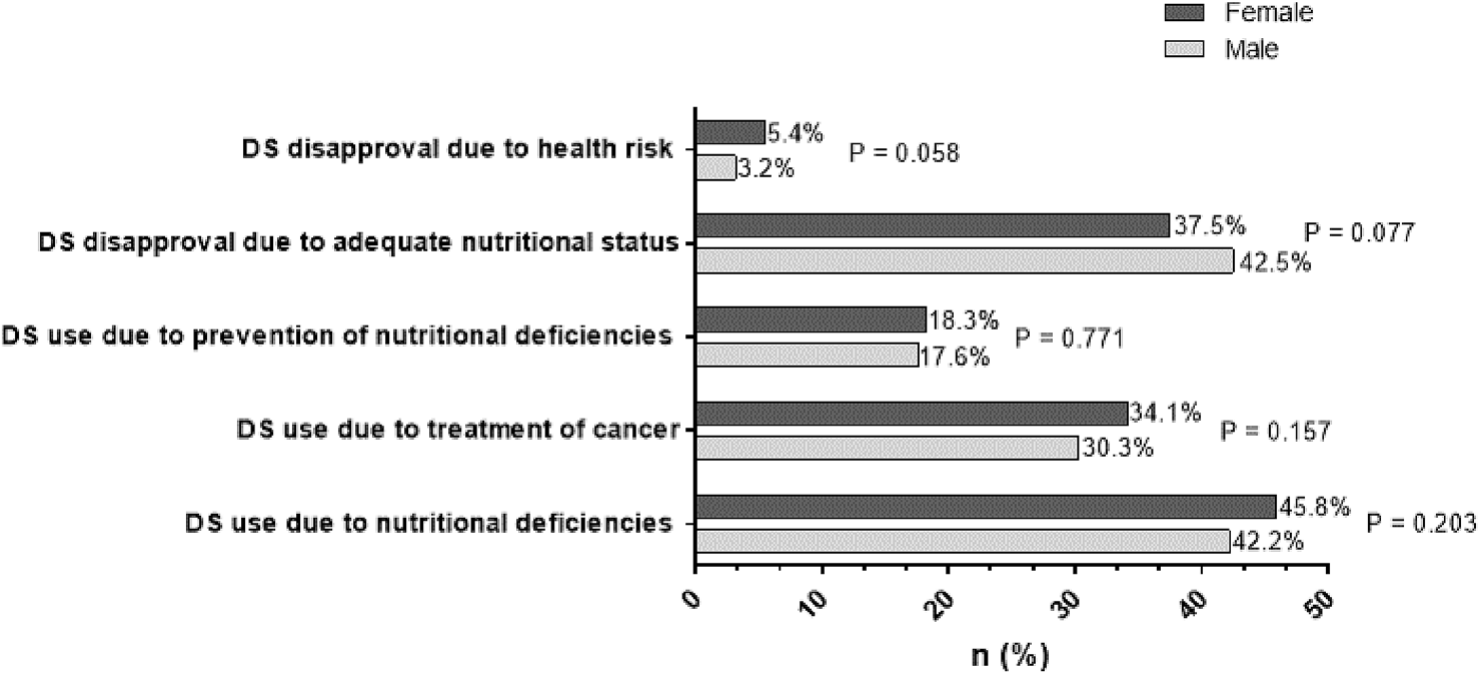
Attitudes towards supplement use in DS users

Pre-formulated statements were used to assess attitude towards DS use in all participants as well as motivation in DS users. Multiple answers were allowed. As can be seen in Fig. 1, the most frequent attitude was to treat nutritional deficiencies, whereas disapproval due to health risk concerns was mentioned least.

**Fig. 2:**
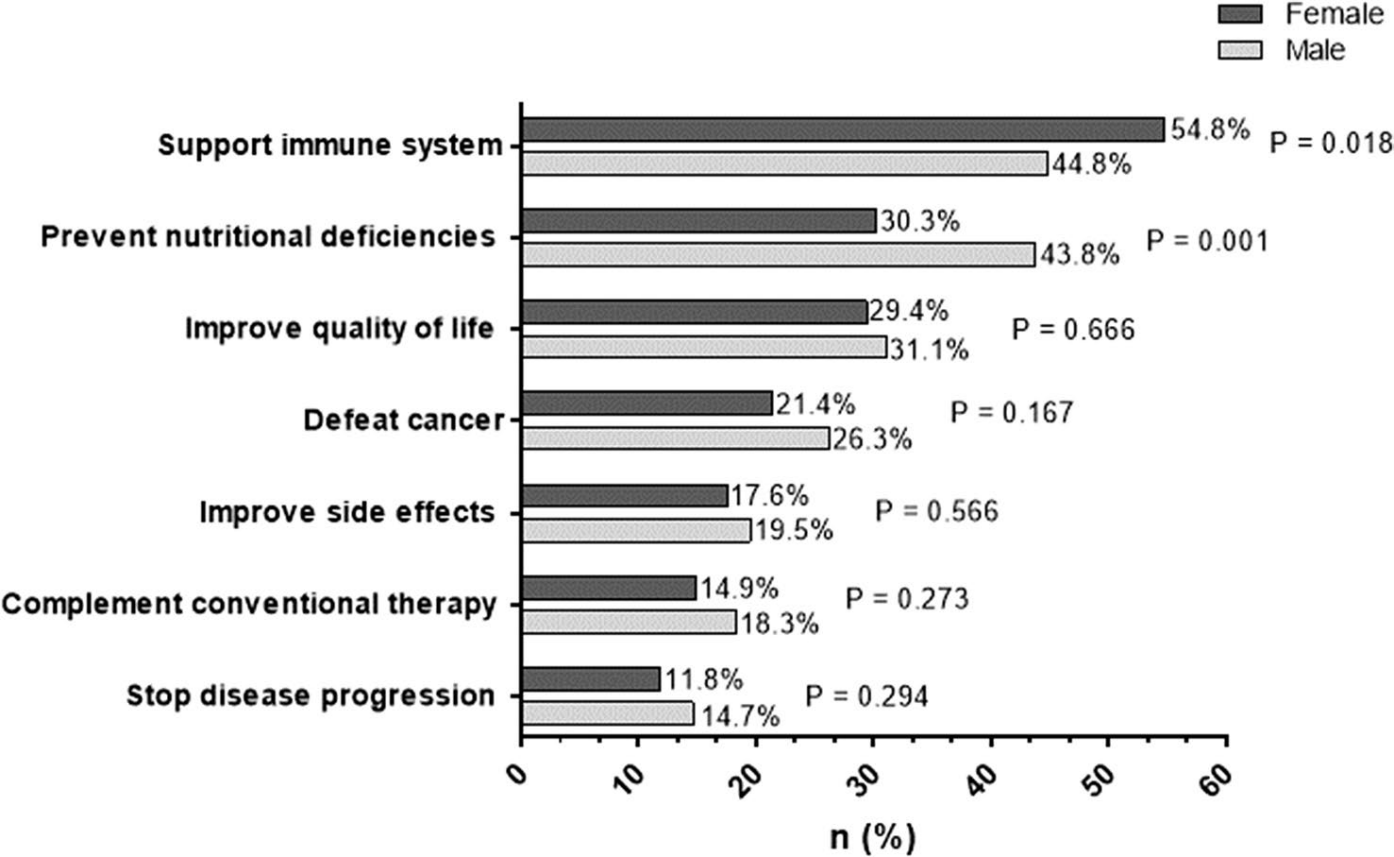
Reasons for dietary supplement use. Multiple answers were allowed

The following statements regarding patients’ motivation were selected in decreasing frequencies: support of the immune system (26.4%), prevention of nutritional deficiencies (18.8%), improvement of quality of life (15.7%), defeat cancer (11.8%), improvement of side effects (9.3%), complement conventional therapy (8.6%) and stop disease progression (6.7%). There was no sex-specific difference except for the statement “support of the immune system” and “prevention of nutritional deficiencies” (see Fig. 2).

### Sources of advice on DS use

In summary, 79.7% of all participants reported having received or acquired information on the influence of nutrition and DS on cancer disease from at least one source whereas approximately a fifth of patients denied receiving any kind of nutritional information. Women (84.8% vs. 74.9%, *p* =  < 0.001) and patients younger than 65 years (84.4% vs. 77.2%, *p* = 0.002) sought advice more often.

Depending on the source of advice, there were differences regarding the use of DS (see Table 4). Patients who acquired information from print media, internet and TV reported using DS significantly more than uninformed patients. The same applies to patients, who were advised by their nutritionist/dietician, pharmacist and homeopath or by other health professionals, whereas no difference was found within the groups of patients, who received advice from their oncologist or physician or those who stated to be uninformed.

**Table 4:** Most frequent sources of advice and DS use

	All *n* = 1217	DS users *n* = 574	Discontinued DS users *n* = 55	Non DS users *n* = 585	*p* value
Source of information, *n* (%)					
Print media	385 (31.6)	214 (37.2)	30 (54.5)	141 (24.1)	< 0.001
Oncologist	362 (29.7)	182 (31.7)	14 (25.5)	166 (28.4)	0.386
Friends or family member	337 (27.7)	194 (33.7)	19 (34.5)	125 (21.4)	< 0.001
Primary care physician	332 (27.3)	161 (28.0)	11 (20.0)	160 (27.4)	0.472
Internet, social media	227 (18.7)	137 (23.9)	18 (32.7)	72 (12.3)	< 0.001
Nutritionist/Dietician	226 (18.6)	127 (22.1)	16 (29.1)	83 (14.2)	< 0.001
TV	222 (18.2)	110 (19.1)	16 (29.1)	96 (16.4)	0.057
Other health care professionals	71 (5.8)	46 (8.0)	3 (5.5)	22 (3.8)	0.007
Homeopath	47 (3.9)	39 (6.8)	3 (5.5)	5 (0.9)	< 0.001
Pharmacist	45 (3.7)	31 (5.4)	2 (3.6)	12 (2.1)	0.009
Other	80 (6.6)	46 (8.0)	5 (9.1)	29 (5.0)	0.074
No information received, *n* (%)	242 (19.9)	74 (12.9)	6 (10.9)	162 (27.7)	< 0.001

Data presented as frequencies (%). *p* values are calculated with Fisher’s exact test. Multiple answers allowed

*DS* dietary supplements

### Changes in dietary habits

**Fig. 3:**
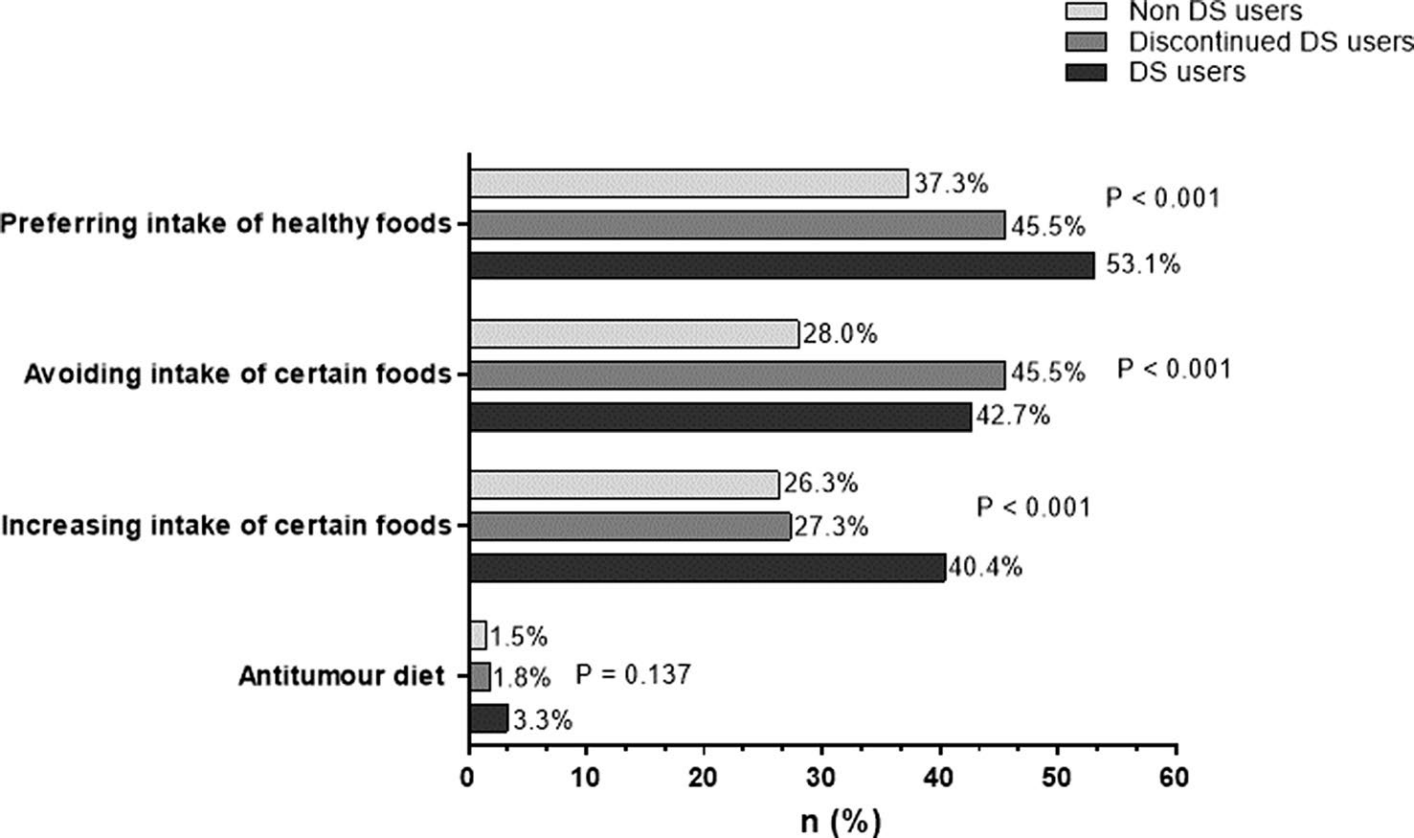
Awareness and changes in dietary habits upon tumour diagnosis. Multiple answers were allowed

Almost half of all participants (49.6%) reported a change of dietary habits upon cancer diagnosis, with the highest percentage in women (55.6% vs. 44.4%, *p* = 0.003). A change of dietary habits was more frequent in patients taking DS (59.9 vs. 39.1%, *p* < 0.001). 33% of participants reported to give preference to certain foods of which fruits, including citrus fruits, were the most frequently mentioned (54.7%). 35.7% of participants reported to avoid certain kinds of food such as meat, alcohol, sugar and fatty foods. Cancer-specific diets did not seem to play a major role as only 2.4% of participants reported following cancer diets (see Fig. 3).

Again, sex-specific differences were found in the group with solid tumor disease (*p* = 0.007). Women with lung cancer (57.1% vs. 42.9%, *p* = 0.033), breast cancer or cancer of the genital tract (53.7% vs. 37.7%, *p* = 0.046), but also with myelodysplastic syndrome (44.4% vs. 19.2%, *p* = 0.049) changed their dietary habits significantly more often than men. Moreover, 58.8% of the participants younger than 65 years reported changes in dietary habits compared to 44% in the cohort older than 65, (*p* =  < 0.001). Patients with solid tumor disease (55.3%, *p* =  < 0.001) and patients with a history of oncological medication (52.4%, *p* = 0.004) changed their dietary habits more often, whereas marital status only had a borderline effect (single 53.8% vs. 47.7%, *p* = 0.050). No difference was identified between changes of dietary habits and duration of disease (*p* = 0.367), academic degree (*p* = 0.339), smoking status (*p* = 0.574) and tumor stage, respectively (*p* = 0.766). Figure 3 shows changes of dietary habits since diagnosis.

## Discussion

In the context of the growing popularity of DS such as micronutrients as well as complementary and alternative medicine related supplements (e.g. herbal and botanical supplements) on the one hand and increasing evidence of potential toxic side effects due to pharmacokinetic interactions on the other hand, assessing dietary supplement use has become one focus in research, but not yet in clinical practice. Our study revealed that almost every second ambulatory patient with cancer used DS which is similar to previous studies. The majority of patients stated to have received or acquired advice on the intake of DS and the most frequently given reason for DS intake was to support the immune system, followed by prevention of nutritional deficiencies.

Data on the prevalence of DS use among ambulatory patients with cancer is limited in number and studies are heterogeneous regarding patient cohorts and design (Holzapfel et al. 2020; Huebner et al. 2014; Konig et al. 2016; Maschke et al. 2017). The majority of data is acquired from patients suffering from breast cancer (Drozdoff et al. 2018; Fremd et al. 2017) and studies are mainly conducted in cooperation with university-based cancer centers. We, therefore, focused on ambulatory patients with cancer who have no regular access to dietary counselling. Our findings reveal that 51.7% of ambulatory patients use, or have used DS. Similar results in patients with cancer have been described elsewhere (Alsanad et al. 2016; Jermini et al. 2019; Luo and Asher 2018; Schuerger et al. 2019). In accordance to some studies (Alsanad et al. 2016; Friedman et al. 2019; Jermini et al. 2019; Konig et al. 2016; Schuerger et al. 2019), our data shows a significantly higher frequency of DS use among women. This effect was due to the cohort with breast- and genital cancer disease, and most likely due to women with breast cancer, who have previously been shown to be highly motivated to use DS (Ferrucci et al. 2009; Holzapfel et al. 2020; Luo and Asher 2018; Miller et al. 2009). In fact, our study results show that overall frequency regarding DS use remains as high as in healthy individuals (Bailey et al. 2011; Friedman et al. 2019; Li et al. 2010) and is only exceeded by the groups of cancer survivors and patients with breast cancer (Drozdoff et al. 2018; Ferrucci et al. 2009; Miller et al. 2009).

Calcium and magnesium are the most frequently taken supplements in our survey, which is in accordance with other studies with cancer patients (Alsanad et al. 2016; Friedman et al. 2019; Wilkinson and Stevens 2014). One-fifth of our participants reported taking either multivitamins or vitamin C, which again is comparable to the previously mentioned studies (Alsanad et al. 2016; Wilkinson and Stevens 2014). Use of herbal and botanical supplements was reported in 12.6% of participants, which is in range with other cross-sectional cancer patient studies (Molassiotis et al. 2005; Wilkinson and Stevens 2014). Besides other non-vitamin-non-mineral DS, especially high dose herbal and botanical supplements are suspected to harbour a higher risk for drug interactions and therefore should not be generally recommended (Caccialanza et al. 2016; Daenen et al. 2015; Frenkel et al. 2013; Hsieh et al. 2014; Mazzanti et al. 2015, 2009).

Due to the fact that neither quality nor frequency of dietary information could be objectified, conclusions regarding the influence of nutritional information on patients´ motivation to use DS have to be drawn carefully. We identified an association between DS use and information by semi-professional sources and lay sources, but also by nutritionists and pharmacists. This finding may be biased by the fact that patients who are actively seeking advice may be more willing to use DS than others and vice versa. Nevertheless, these findings raise the question about the quality in terms of evidence-based information on DS use provided by health professionals and highlight the need for further training and education regarding this issue. Research groups like the Competence Network Complementary Medicine in Oncology (KOKON) funded by the German Cancer Aid Society and others, (Guthlin et al. 2020; He et al. 2019; Helmer et al. 2019; Ziemann et al. 2019) are currently working on this issue to implement evidence-based information on DS and nutrition and other aspects of complementary medicine into patient-doctor-communication to prevent harm and improve treatment compliance and tolerability (Frenkel et al. 2013; Greenlee et al. 2017; Shalom-Sharabi et al. 2017).

Almost half of the respondents reported to have changed their dietary habits and wished to eat a „healthier “ diet. Participants reported eating more vegetables and fruits and to avoid certain foods. With respect to the well-known toxic interactions between certain kinds of citrus fruits, e.g. grapefruits, bitter oranges and pomegranates, and a growing range of pharmaceuticals, these findings again underline the need for a detailed exploration of dietary concerns (de Jong et al. 2015).

Our data is subject to limitations due to the use of a self-reported questionnaire which harbours the well-known risk of under- or overestimation. We also cannot exclude inclusion bias associated with the type of recruitment (voluntary). Being aware of study-derived estimates of up to 75% of patients who refrain from disclosing DS use to their health provider for different reasons, we used a self-administered questionnaire to assure participants would feel free to answer without other influence (Levy et al. 2018). As a consequence, there was no chance to discuss missing or ambiguous data with the patient, which also may contribute to false estimation.

## Conclusion

As almost every second patient with cancer reported changing their dietary habits and using DS, our findings emphasize the need to implement nutritional consultation routinely into cancer care in the ambulatory setting, as well as to meet patients’ needs, and to prevent potential interactions with anticancer therapies.

## Data Availability

Patients’ data and material belong to the Charité—Universitätsmedizin Berlin, corporate member of Freie Universität Berlin, Humboldt—Universität zu Berlin and access is restricted according to data protection laws. The datasets are available from the corresponding author on reasonable request.

## References

[CR1] Alsanad SM, Howard RL, Williamson EM (2016). An assessment of the impact of herb-drug combinations used by cancer patients. BMC Complement Altern Med.

[CR2] Bailey RL (2011). Dietary supplement use in the United States, 2003–2006. J Nutr.

[CR3] Caccialanza R (2016). Nutritional support in cancer patients: a position paper from the Italian Society of Medical Oncology (AIOM) and the Italian Society of Artificial Nutrition and Metabolism (SINPE). J Cancer.

[CR4] Daenen LG (2015). Increased plasma levels of chemoresistance-inducing fatty acid 16:4(n-3) after consumption of fish and fish oil. JAMA Oncol.

[CR5] Davis EL, Oh B, Butow PN, Mullan BA, Clarke S (2012). Cancer patient disclosure and patient-doctor communication of complementary and alternative medicine use: a systematic review. Oncologist.

[CR6] de Jong J (2015). Effect of CYP3A perpetrators on ibrutinib exposure in healthy participants. Pharmacol Res Perspect.

[CR7] Drozdoff L, Klein E, Kiechle M, Paepke D (2018). Use of biologically-based complementary medicine in breast and gynecological cancer patients during systemic therapy. BMC Complement Altern Med.

[CR8] Du M (2020). Dietary supplement use among adult cancer survivors in the United States. J Nutr.

[CR9] Ferrucci LM, McCorkle R, Smith T, Stein KD, Cartmel B (2009). Factors related to the use of dietary supplements by cancer survivors. J Altern Complement Med.

[CR10] Fremd C (2017). Use of complementary and integrative medicine among German breast cancer patients: predictors and implications for patient care within the PRAEGNANT study network. Arch Gynecol Obstet.

[CR11] Frenkel M (2013). Integrating dietary supplements into cancer care. Integr Cancer Ther.

[CR12] Friedman J, Birstler J, Love G, Kiefer D (2019). Diagnoses associated with dietary supplement use in a national dataset. Complement Ther Med.

[CR13] Greenlee H (2017). Clinical practice guidelines on the evidence-based use of integrative therapies during and after breast cancer treatment. CA Cancer J Clin.

[CR14] Guthlin C (2020). KOKON: A Germany-Wide Collaborative Research Project to identify needs, provide information, foster communication and support decision-making about complementary and alternative medicine in oncology. Complement Med Res.

[CR15] He X (2019). ALOHA: developing an interactive graph-based visualization for dietary supplement knowledge graph through user-centered design. BMC Med Inform Decis Mak.

[CR16] Helmer SM, Rogge AA, Fischer F, Pach D, Horneber M, Roll S, Witt CM (2019). Evaluation of a blended-learning training concept to train oncology physicians to advise their patients about complementary and integrative medicine (KOKON-KTO): study protocol for a prospective, multi-center, cluster-randomized trial. Trials.

[CR17] Holzapfel CKA, Jaeckel B, Martignoni M, Hauner D, Hauner H (2020) Ernährungsformen und Einnahme von Nahrungsergänzungsmitteln bei Patienten mit Tumorerkrankungen

[CR18] Horneber M, van Ackeren G, Fischer F, Kappauf H, Birkmann J (2018). Addressing unmet information needs: results of a clinician-led consultation service about complementary and alternative medicine for cancer patients and their relatives. Integr Cancer Ther.

[CR19] Hsieh YW, Huang CY, Yang SY, Peng YH, Yu CP, Chao PD, Hou YC (2014). Oral intake of curcumin markedly activated CYP 3A4: in vivo and ex-vivo studies. Sci Rep.

[CR20] Huebner J (2014). User rate of complementary and alternative medicine (CAM) of patients visiting a counseling facility for CAM of a German comprehensive cancer center. Anticancer Res.

[CR21] Jermini M (2019). Complementary medicine use during cancer treatment and potential herb-drug interactions from a cross-sectional study in an academic centre. Sci Rep.

[CR22] Konig J, Geschwill K, Lang A, Tauchert FK, Hofheinz RD, Kripp M (2016). Use of complementary and alternative medicine in cancer patients: a prospective questionnaire-based study in an oncological outpatient. Clinic Oncol Res Treat.

[CR23] Levy AG, Scherer AM, Zikmund-Fisher BJ, Larkin K, Barnes GD, Fagerlin A (2018). Prevalence of and factors associated with patient nondisclosure of medically relevant information to clinicians. JAMA Netw Open.

[CR24] Li K, Kaaks R, Linseisen J, Rohrmann S (2010). Consistency of vitamin and/or mineral supplement use and demographic, lifestyle and health-status predictors: findings from the European Prospective Investigation into Cancer and Nutrition (EPIC)-Heidelberg cohort. Br J Nutr.

[CR25] Luo Q, Asher GN (2018). Use of dietary supplements at a comprehensive cancer center. J Altern Complement Med.

[CR26] Maschke J, Kruk U, Kastrati K, Kleeberg J, Buchholz D, Erickson N, Huebner J (2017). Nutritional care of cancer patients: a survey on patients' needs and medical care in reality. Int J Clin Oncol.

[CR27] Mazzanti G, Menniti-Ippolito F, Moro PA, Cassetti F, Raschetti R, Santuccio C, Mastrangelo S (2009). Hepatotoxicity from green tea: a review of the literature and two unpublished cases. Eur J Clin Pharmacol.

[CR28] Mazzanti G, Di Sotto A, Vitalone A (2015). Hepatotoxicity of green tea: an update. Arch Toxicol.

[CR29] Miller PE, Vasey JJ, Short PF, Hartman TJ (2009). Dietary supplement use in adult cancer survivors. Oncol Nurs Forum.

[CR30] Molassiotis A (2005). Use of complementary and alternative medicine in cancer patients: a European survey. Ann Oncol.

[CR31] Schuerger N, Klein E, Hapfelmeier A, Kiechle M, Brambs C, Paepke D (2019). Evaluating the demand for integrative medicine practices in breast and gynecological cancer patients. Breast Care (Basel).

[CR32] Shalom-Sharabi I, Lavie O, Samuels N, Keinan-Boker L, Lev E, Ben-Arye E (2017). Can complementary medicine increase adherence to chemotherapy dosing protocol? A controlled study in an integrative oncology setting. J Cancer Res Clin Oncol.

[CR33] Wilkinson JM, Stevens MJ (2014). Use of complementary and alternative medical therapies (CAM) by patients attending a regional comprehensive cancer care centre. J Complement Integr Med.

[CR34] Ziemann J, Lendeckel A, Muller S, Horneber M, Ritter CA (2019). Herb-drug interactions: a novel algorithm-assisted information system for pharmacokinetic drug interactions with herbal supplements in cancer treatment. Eur J Clin Pharmacol.

